# A novel threefold interpenetrated zirconium metal–organic framework exhibiting separation ability for strong acids†

**DOI:** 10.1039/d3sc04171a

**Published:** 2024-01-02

**Authors:** Kyoko Shiraishi, Kazuya Otsubo, Kenichi Kato, Masaaki Sadakiyo

**Affiliations:** a Department of Applied Chemistry, Faculty of Science Division I, Tokyo University of Science 1-3 Kagurazaka Shinjuku-ku Tokyo 162-8601 Japan sadakiyo@rs.tus.ac.jp; b Department of Chemistry, Faculty of Science Division I, Tokyo University of Science 1-3 Kagurazaka Shinjuku-ku Tokyo 162-8601 Japan; c RIKEN SPring-8 Center Sayo-gun Hyogo 679-5148 Japan

## Abstract

We report on the synthesis and selective adsorption property of a novel threefold interpenetrated Zr-based metal–organic framework (MOF), [Zr_12_O_8_(OH)_8_(HCOO)_15_(BPT)_3_] (BPT^3−^ = [1,1′-biphenyl]-3,4′,5-tricarboxylate) (abbreviated as Zr-BPT). This MOF shows a high tolerance to acidic conditions and has permanent pores, the size of which (approx. <5.6 Å) is the smallest ever reported among porous Zr-based MOFs with high acid tolerance. Zr-BPT selectively adsorbs aryl acids due to its strong affinity for them and exhibits separation ability, even between strong acid molecules, such as sulfonic and phosphonic acids. This is the first demonstration of a MOF exhibiting selective adsorption and separation ability for strong acids.

## Abstract 2

A novel threefold interpenetrated Zr-based MOF, Zr-BPT, which exhibits high acid tolerance and has very small permanent pores, was created. It selectively adsorbs aryl acids even between strong acid molecules, such as sulfonic and phosphonic acids.

## Graphical abstract

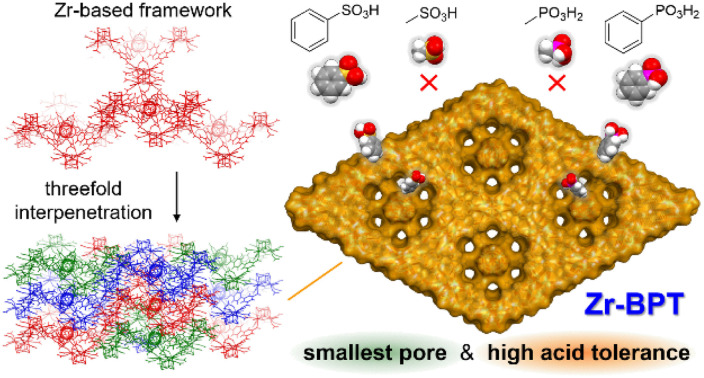

## Introduction

The development of novel architectures of porous metal–organic frameworks (MOFs) and exploring their functionalities have attracted great interest over the past two decades.^1,2^ In particular, MOFs have been intensively studied as separating materials—they exhibit various selective adsorption properties because of their designable pores, with strong interactions or well-matched sizes for specific guest molecules.^3–6^ To date, numerous examples of selective adsorption or separation by MOFs have been reported; however, almost all the cases have only dealt with neutral molecules (not acids or bases) as the adsorbates, which would be mainly due to the problem in their stability. Not many studies have been carried out on the selective adsorption behaviour or separation ability of MOFs for acidic or basic molecules. In particular, there is no report of MOFs showing separation or adsorption selectivity among strong acids such as sulfonic and phosphonic acids, while these species are considered important in chemistry.^7,8^

Our focus was on creating a novel MOF exhibiting selective adsorption properties and separation ability for strong acids. For the design of such a novel MOF, there are two great hurdles to overcome. The first problem is the stability of MOFs as mentioned—they are normally unstable under acidic conditions, while some hydrolytically stable MOFs have been reported.^9,10^ Regarding this point, recent research has fortunately shown an important way in which the use of Zr^4+^ ions (as central metals) with carboxylate ligands tends to afford MOFs (*i.e.*, Zr-based MOFs) that exhibit excellent stability to acids.^11–14^ The second problem lies in the pore size of Zr-based MOFs. Almost all Zr-based MOFs have large-sized pores (>8 Å). This is possibly due to the relatively inflexible secondary building units composed of six-membered Zr^4+^ (Zr_6_) clusters^11–14^ that prevent diminishing of the pore size by local distortion of the framework for strong binding of included guest molecules. MOFs with large-sized pores are indeed important in storage materials.^15^ However, from the point of view of selective adsorption, it must be a disadvantage for the recognition of small guest molecules because the strong binding of specific molecules should occur through multipoint interactions with the MOF's framework inside limited-sized pores.^16,17^

**Fig. 1:**
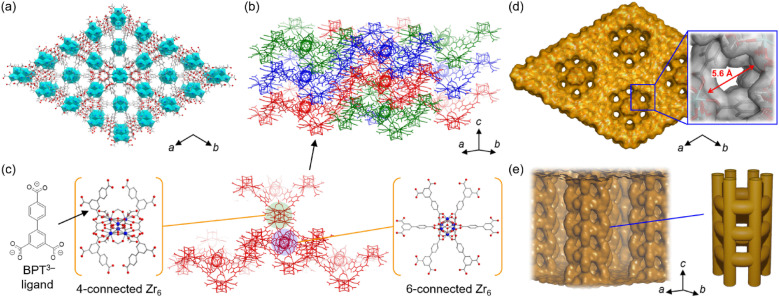
Representations of the crystal structure of Zr-BPT. (a) View along the *c*-axis. Light gray, gray, and red colors correspond to hydrogen, carbon, and oxygen atoms, respectively. The coordinated Zr^4+^ is shown as a polyhedron. (b) The threefold interpenetrated structure of the framework. Each independent infinite framework is described with a different color. (c) An independent infinite framework composed of two types of Zr_6_ clusters. (d) Illustration of the pore surface along the *c*-axis (solvent-accessible area with a 1.40 Å probe radius). The inset describes a magnified view with the van der Waals surface (gray color) to show the maximum diameter of the 1D pore along the *c*-axis. (e) Side view of the inner 1D pore along the *c*-axis (solvent-accessible area with a 1.40 Å probe radius) and its schematic illustration. The framework was omitted for clarity.

Here, we report on the creation of a novel MOF exhibiting selective adsorption of specific acidic molecules and separation ability for strong acids such as sulfonic and phosphonic acids. To construct small-sized pores, even with the rigid Zr-based framework, we tried to introduce an interpenetrating structure by employing an elongated carboxylate ligand, [1,1′-biphenyl]-3,4′,5-tricarboxylate (BPT^3−^). By employing this ligand, we succeeded, for the first time, in constructing a threefold interpenetrated Zr-based MOF, [Zr_12_O_8_(OH)_8_(HCOO)_15_(BPT)_3_] (abbreviated as Zr-BPT) (Fig. 1), while a few examples of twofold interpenetrated Zr-based MOFs have been reported previously.^18,19^ This novel MOF shows high tolerance to acidic conditions and has small-sized pores (approx. <5.6 Å) due to the threefold interpenetrated structure. It selectively adsorbs strong aryl acids rather than strong alkyl acids and thus exhibits separation ability, even for strong acids.

## Experimental

### Synthesis of H_3_BPT ([1,1′-biphenyl]-3,4′,5-tricarboxylic acid)

The ligand H_3_BPT was synthesized according to a previous report.^20^ Dehydrated tetrahydrofuran (THF) (270 mL) and dehydrated ethanol (120 mL) were added to 18-crown-6-ether (2.00 g, 7.56 mmol) under nitrogen. After bubbling nitrogen gas for 60 min, dimethyl 5-bromoisophthalate (8.17 g, 29.9 mmol), 4-(ethoxycarbonyl)phenylboronic acid (6.96 g, 35.9 mmol), [1,1′-bis(diphenylphosphino)ferrocene]dichloropalladium(ii) (0.55 g, 0.75 mmol), 2-dicyclohexylphosphino-2′,6′-dimethoxybiphenyl (0.62 g, 1.5 mmol), and K_3_PO_4_ (12.72 g, 59.9 mmol) were added and the mixture stirred for 22 h at 60 °C under nitrogen. The resulting mixture was then filtered through celite and the solvent removed by evaporation. The target intermediate, an ester of H_3_BPT, was separated from the residue using column chromatography (hexane : ethyl acetate = 3 : 1); a yellow oil was obtained. This intermediate was mixed with THF (150 mL), methanol (150 mL), and 0.185 M sodium hydroxide aqueous solution (150 mL). The mixture was heated and stirred at 50 °C for 13 h. The solvent of the resulting solution was then removed by evaporation. The crude product was diluted with water and then extracted with ethyl acetate three times. The aqueous layer was acidified to pH 1 with 3 M HCl. The precipitate was filtered, washed with water, and dried under vacuum at 220 °C (yield: 7.18 g, 84%). ^1^H NMR (DMSO-d6): 8.46 (s), 8.40 (s), 8.03 (d), and 7.86 (d) ppm.

### Synthesis of Zr-BPT⊃(Guest)_*n*_

The target MOF was synthesized using a solvothermal method. ZrCl_4_ (70 mg, 0.30 mmol) and H_3_BPT (29 mg, 0.10 mmol) were stirred in a mixed solvent of *N*,*N*-dimethylformamide (DMF) and formic acid (volumetric ratio 1 : 1) (3.0 mL) for 20 min at room temperature (RT) in a Teflon reactor. The reactor was then sealed and the content heated at 150 °C for 48 h, affording a white precipitate. The precipitate was collected by filtration. It was immersed in DMF for 3 days at RT (during washing, the solvent was replaced daily). After filtration, a white powder was obtained; it was dried in the air at RT (yield: 82 mg, 23%). Elemental analysis: calcd (for [Zr_12_O_8_(OH)_8_(HCOO)_15_(C_15_H_7_O_6_)_3_](C_3_H_7_NO)_9.0_(H_2_O)_5.3_): C 28.73%, H 3.26%, N 3.47%; found: C 28.73%, H 3.19%, N 3.58%.

### Synthesis of MOF-808⊃(Guest)_*n*_ ([Zr_6_O_4_(OH)_4_(HCOO)_6_(BTC)_2_]⊃(Guest)_*n*_ (BTC^3−^ = 1,3,5-benzenetricarboxylate))

MOF-808, which was used for comparison of adsorption experiments as described below, was synthesized using a solvothermal method according to a previous report.^21^ ZrOCl_2_·8H_2_O (1.7 g, 5.3 mmol) and trimesic acid (0.37 g, 1.8 mmol) were stirred in a mixed solvent (DMF : formic acid = 1 : 1) (200 mL) for 1 h at RT and then heated at 70 °C for 36 h to give a white precipitate. The precipitate was filtered and washed in DMF and acetone for 3 days (during washing, the solvent was replaced daily). After filtration, a white powder was obtained; it was dried in the air at RT (yield: 0.83 g, 22%). Elemental analysis: calcd (for [Zr_6_O_4_(OH)_4_(HCOO)_6_(C_9_H_3_O_6_)_2_](C_3_H_7_NO)_6.4_(H_2_O)_15.9_): C 24.50%, H 4.41%, N 4.23%; found: C 24.21%, H 4.11%, N 4.64%.

### Stability tests for acids or bases

Activation of Zr-BPT was performed under the optimal conditions (130 °C overnight under vacuum, which was determined by N_2_ adsorption experiments described below) before any adsorption experiments described below. Zr-BPT was immersed in each solution (aqueous solution of 1.0 M HCl (pH = 0), 0.010 M NaOH (pH = 12), and 0.10 M NaOH (pH = 13)) for 24 h. Thereafter, powder samples were collected by filtration and then dried under vacuum at RT. The obtained samples were investigated using X-ray powder diffraction (XRPD) measurements.

### Screening of acid adsorption

Zr-BPT (34.6 mg, 0.01 mmol) was immersed in 50.0 mM aqueous solutions (50.0 mL) of each acidic molecule (all of the used acids are demonstrated in Fig. 4 and S16†) for a day at 298 K. After removing Zr-BPT, the adsorption quantity (*q*_acids_) was calculated from the concentration of the acids in the supernatant solution. Determination was carried out using ^1^H NMR and the following equation
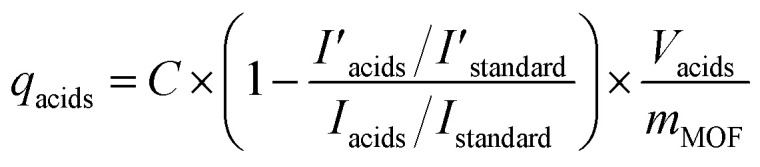
where *C*, *I*_acid_
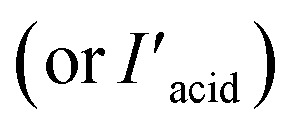
, *I*_standard_
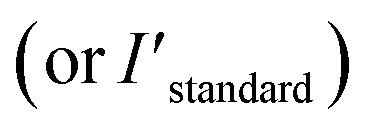
, *V*_acids_, and *m*_MOFs_ correspond to the initial concentration of the adsorbate (50.0 mM), area of the observed peak from the acid molecule (*I*_acid_: before adsorption, 
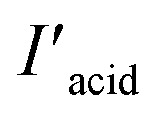
: after adsorption), area of the observed peak from the standard acetonitrile (MeCN) molecule (*I*_standard_: before adsorption, 
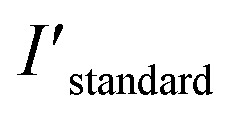
: after adsorption), volume of the acidic solution used for the adsorption experiment (50.0 mL), and weight of the MOF adsorbent, respectively. A 50.0 mM MeCN solution (dissolved in DMSO-d6) was used as the standard. ^1^H NMR spectra were recorded for a solution comprising 100 μL of the standard solution, 100 μL of the sample solution, and 300 μL of DMSO-d6 solvent (*e.g.* Fig. S9†).

### Adsorption isotherms for acid molecules measured in solution phase

Zr-BPT (34.6 mg, 0.01 mmol) or MOF-808 (17.1 mg, 0.01 mmol) was immersed in an aqueous solution (50.0 mL) of each acidic substance (5.0, 10.0, 20.0, 30.0, 50.0, 100 mM) for a day at 298 K (activation of MOF-808 was performed at 150 °C for a night under vacuum before any adsorption experiments described below). After removing the MOF samples, the adsorption quantity (*q*_acids_) was calculated from the concentration of the acids in the supernatant solution, which was determined using ^1^H NMR, as described in the case of the screening process.

### Separation experiments with mixed solution of strong acids

Zr-BPT (34.6 mg, 0.01 mmol) or MOF-808 (17.1 mg, 0.01 mmol) was immersed in an aqueous solution (50.0 mL) containing both aryl (benzenesulfonic acid (BS) or phenylphosphonic acid (PP)) and alkyl acids (methanesulfonic acid (MS) or methylphosphonic acid (MP)) (5.0, 10.0, 20.0, 30.0, 50.0, 100 mM for each substance) for a day at 298 K. After removing the MOF, the concentration of the acid substances in the supernatant solution was determined using ^1^H NMR, as described in the case of the screening process. Excess percentage for the aryl acid (BS or PP) was calculated using the following equation
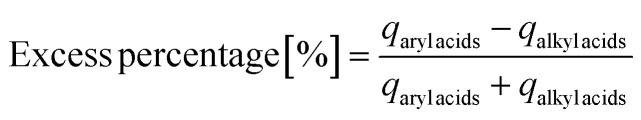
where *q*_aryl acids_ and *q*_alkyl acids_ correspond to the adsorption quantity of aryl acids (BS or PP) and alkyl acids (MS or MP), respectively.

### Physical measurements

XRPD measurements were performed in air at RT using a MiniFlex600 (Cu-Kα) instrument (Rigaku, Inc.). Synchrotron XRPD measurements were performed at RT on the RIKEN Materials Science I Beam Line BL44B2 located at SPring-8 (*λ* = 0.80010 or 0.79973 Å).^22^ Powder samples in different states were sealed in a glass capillary (borosilicate, 0.5 mm diameter; WJM-Glas Müller GmbH). For the Rietveld refinement, the sample was preliminarily activated at 130 °C for a night under vacuum to remove guest molecules inside the pores (the same conditions were applied for the adsorption measurements). For the measurements of the samples after acid adsorption of the strong acid (*i.e.*, 0.01–0.05 BS, in Fig. S12†), they were sealed under vacuum at RT. The solvent-accessible area inside Zr-BPT was calculated and demonstrated using BIOVIA Discovery Studio software. A possible adsorption site for the guests was identified using the Adsorption Locator module in BIOVIA Materials Studio software. Thermogravimetry (TG) analysis was performed using a Thermo Plus Evo2 TG-DTA8122 (Rigaku, Inc.) with a heating rate of 5 °C min^−1^ under N_2_ gas flow (100 mL min^−1^). Fourier transformed infrared (IR) spectra were collected through an attenuated toral reflection (ATR) method using FT/IR4600 (JASCO, Inc.) at RT. Scanning electron microscope (SEM) images were collected using a JSM-7001F/SHL instrument (JEOL, Inc.) with an acceleration voltage of 17.7 kV. Nitrogen adsorption measurements were performed at 77 K using BELSORP-max (Microtrac BEL, Inc.) and TriStar II 3020 (Micromeritics, Inc.) instruments. Prior to carrying out measurements, the samples were activated at 130 °C under vacuum overnight. The activation temperature was optimized according to the results of N_2_ adsorption measurements (*i.e.*, the adsorption amount of N_2_) carried out using samples activated at various temperatures. Analyses for Brunauer–Emmett–Teller (BET) surface area and pore size distribution carried out with Grand Canonical Monte Carlo (GCMC) simulations were performed using BELMaster software. Adsorption isotherms for guest vapors were measured using a BELSORP-max (Microtrac BEL, Inc.). The samples were also activated prior to measurements, as described above. ^1^H NMR spectra were recorded with JNM-ECA500 (JEOL, Inc.) and ADVANCE NEO 400 (Bruker, Inc.) spectrometers at RT. In the case of ^1^H NMR measurements for digested MOF samples, they were digested in a mixed solution of 1.0 M NaOH/D_2_O (300 μL) and DMSO-d6 (200 μL).

### Rietveld refinement for Zr-BPT

The initial structure for Rietveld refinement was solved as follows. Powder indexing and Le Bail refinement were performed using the EXPO2014 program.^23^ Positions of Zr clusters were solved using the charge-flipping method with Superflip^24^ and EDMA.^25^ After Zr clusters were modeled, BPT ligands and formic acid molecules were placed in appropriate positions using Materials Studio 6.0 (Accelrys Software Inc.), which was used for the initial structure for Rietveld refinement. The Rietveld refinement was performed using the program RIETAN-FP.^26^ For structure refinement, soft restraints were applied to all bond lengths, bond angles, and dihedral angles to retain molecular geometry. For the Rietveld refinement, due to the large unit cell and the asymmetric unit, there were a large number of parameters for the profile function (such as atomic coordinates, thermal displacement parameters, and site occupancy factors), which are strongly related to one another. Because of this, we carefully refined each parameter for Rietveld refinement in a gradual manner. Due to a large number of parameters, we used fixed atomic coordinates, site occupancy factors, and thermal displacement parameters at the final stage. The final Rietveld refinement result at 298 K was the following: trigonal, space group *P*3̄1*m* (no. 162), *a* = 26.659(1) Å, *c* = 10.1030(4) Å, *V* = 6218.1(5) Å^3^, *Z* = 2, *R*_wp_ = 7.37%, *R*_e_ = 2.61%, *R*_p_ = 5.17%, *R*_B_ = 4.70%, *R*_F_ = 2.08%, *S* = 2.82, CCDC reference number 2265356.

## Results and discussion

After exploring the reaction conditions, Zr-BPT was successfully synthesized using a typical solvothermal method with ZrCl_4_ as a metal source, H_3_BPT as a ligand, and a mixed solvent of DMF and formic acid. Note that, even after careful optimization of the synthetic procedure, we could not obtain a single crystal, as indicated by a SEM image of the resulting fine powder of Zr-BPT (Fig. S1†). By contrast, this MOF shows an XRPD pattern with sharp peaks, *i.e.*, a crystalline nature of the sample, which is not derived from any of the reagents or by-products (Fig. S2†). Therefore, to determine the crystal structure of Zr-BPT, we performed Rietveld analysis using the pattern obtained from synchrotron XRPD measurements. We succeeded in obtaining a reasonable crystal structure (Fig. 1) that showed excellent agreement with the experimental data (Fig. S3†).

**Fig. 2:**
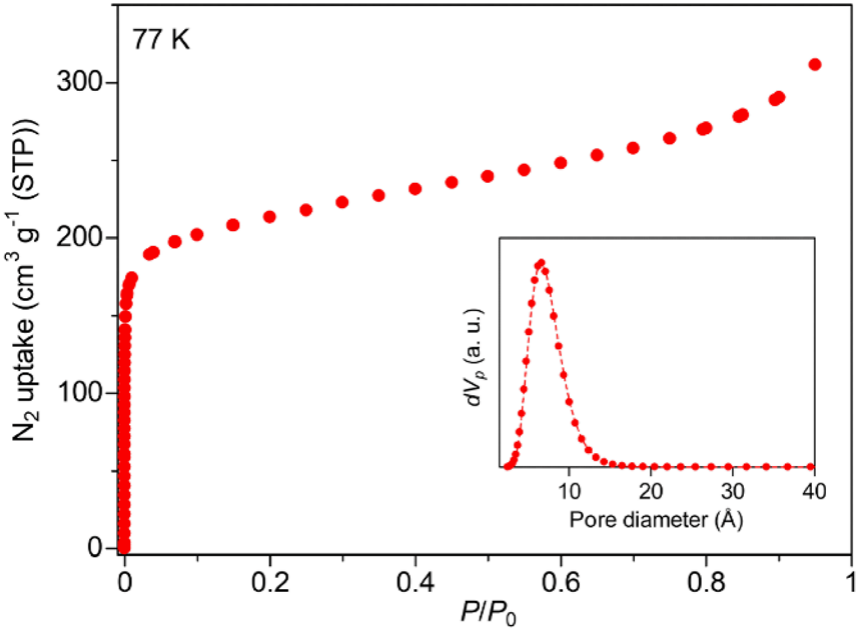
N_2_ adsorption isotherms of Zr-BPT, measured at 77 K. The inset shows the pore size distribution estimated by GCMC analysis.

The framework of Zr-BPT consists of Zr_6_ clusters, connected by deprotonated ligands of BPT^3−^. There are two types of Zr_6_ clusters connecting to four or six BPT^3−^ ligands (Fig. 1c), whereas Zr-based MOFs often have one type of Zr_6_ cluster.^11–14^ The structural comparison and detailed structures of the 4- and 6-connected Zr_6_ clusters in Zr-BPT are shown in Fig. S4.† The 6-connected Zr_6_ cluster in Zr-BPT connects to the BPT^3−^ ligands through a chelate coordination with –COO^−^ groups on 12 coordination sites, resulting in arranging the bridging ligands on the same plane towards six directions. This structure is very similar to the case of a previously reported Zr-based MOF (Zr-BTB).^27^ On the other hand, the 4-connected Zr_6_ cluster in Zr-BPT connects to the ligands through a single coordination with –COO^−^ groups on 4 coordination sites, which is not similar to the previous case (NU-1400).^28^ The independent three-dimensional framework constructs a threefold interpenetrated structure (Fig. 1b and c), resulting in small-sized one-dimensional (1D) pores along the *c*-axis (Fig. 1d), which partly connect to neighboring channels (Fig. 1e). Although it is not easy to express the diameter of this 1D pore, because of its complicated shape, we could state that the diameter of the 1D pore is approx. <5.6 Å, which is the diameter at the largest area of the pores along the *c*-axis (Fig. 1d). The porous character of Zr-BPT was confirmed by N_2_ adsorption measurements at 77 K. As shown in Fig. 2, the large amount of adsorption in the low-pressure region clearly confirmed the porous nature of the sample. The BET surface area was estimated to be 777 m^2^ g^−1^ and the average diameter of the micropores in the MOF was estimated to be 5.8 Å through GCMC analysis (Fig. 2), which is consistent with the results of the structural analysis presented above.

Fig. S5† shows the IR spectra of Zr-BPT. The absence of the C=O stretching mode of the carboxylic acid group (at 1733 cm^−1^ on H_3_BPT) and the presence of the C=O stretching mode of carboxylate (at 1651 cm^−1^) and Zr–O stretching mode (642 cm^−1^) in Zr-BPT clearly indicated the successful formation of the MOF through the coordination bond, which is consistent with the structural analysis. Fig. S6† shows a TG curve of Zr-BPT. The weight loss due to the included water molecules was observed below 100 °C (10% weight loss, corresponding to approximately 18H_2_O molecules per formula unit). The large weight loss, which is attributable to thermal decomposition or desorption of organic components, was observed at around 140–350 °C. Approximately 23% weight loss of this region would be derived from desorption (or decomposition) of 15 formate ions included in the sample (calculated to be 22% weight loss). We also evaluated thermal stability of the framework by measuring XRPD patterns at various temperatures. As shown in Fig. S7,† the crystallinity of Zr-BPT (under vacuum) remained below 250 °C and the framework collapsed above 300 °C, indicating its moderate thermal stability as a MOF.

**Fig. 3:**
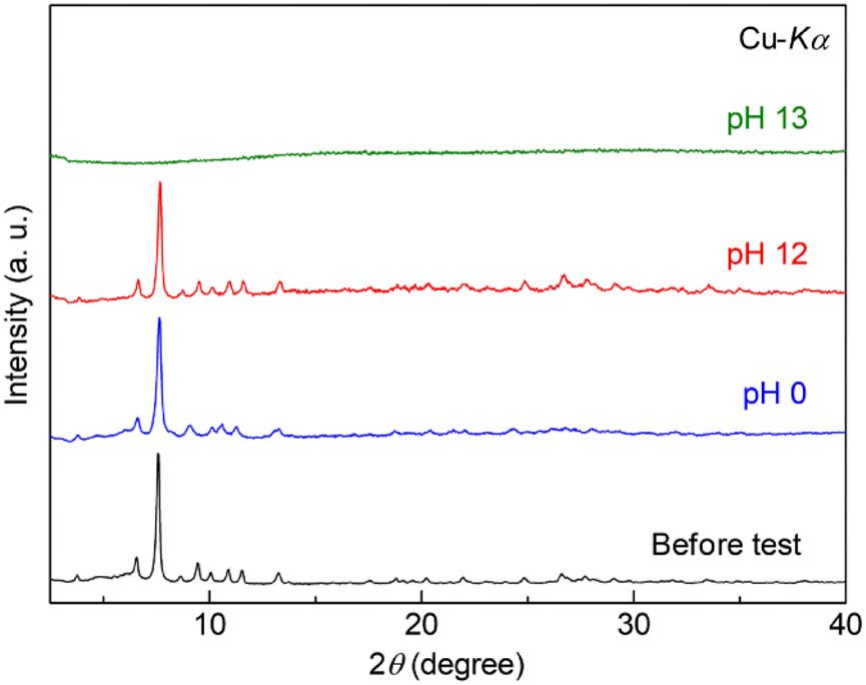
XRPD patterns of Zr-BPT before and after the stability tests under acidic or basic conditions.

The tolerance of Zr-BPT to acids or bases was also tested by exposing a sample in solution to various pH conditions at RT. After exposure in the pH range of 0–12, there was no change in the XRPD patterns (Fig. 3). This indicated high tolerance of Zr-BPT to acids, similar to some other Zr-based MOFs.^11–14^ To the best of our knowledge, the pore of Zr-BPT is the smallest inner micropore among Zr-based MOFs with both high acid tolerance and apparent porosity.

Note that it is known that Zr-based MOFs become defective after exposure to acids, while their crystallinity remains.^29^^1^H NMR spectra of the digested Zr-BPT (Fig. S8†) after exposure to the acid solution (pH = 0) indicated the apparent decrease of the amount of formate ions. The number of formate ions in Zr-BPT is variable in the range from 2 to 15 per formula unit, which would result in forming some of the defects, *i.e.*, OH^−^ or H_2_O instead of the coordinating formate ions. However, as discussed later, the adsorption ability of the non-defective sample for the specific target molecules (*i.e.*, aryl acids) is remarkably higher than that of the defective Zr-BPT. Therefore, we used untreated Zr-BPT for the following adsorption experiments.

**Fig. 4:**
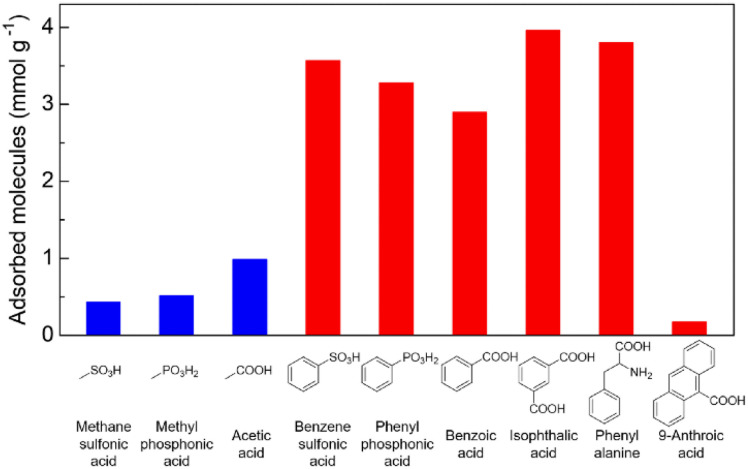
Amounts of adsorbed acidic molecules in Zr-BPT at 298 K.

To determine the selective adsorption property of Zr-BPT for specific acidic molecules, first, we performed adsorption experiments by soaking a sample in aqueous solutions of various acid molecules, such as sulfonic, phosphonic, and carboxylic acids, with different functional groups, at 298 K, for screening purposes. As shown in Fig. 4, there is a clear tendency that Zr-BPT preferably adsorbs aryl acids rather than alkyl acids, in all cases (sulfonic, phosphonic, and carboxylic acids), implying the existence of hydrophobic interactions between the guest molecules and framework. By contrast, there is almost no adsorption of anthracene-9-carboxylic acid, although it has a large hydrophobic part of aromatic rings. This suggests that there is some upper limit to the size of the guest molecules for adsorption, because of the small-sized pores. Results clearly indicated that Zr-BPT has the potential to show selective adsorption behaviour or separation ability even for strong acid molecules. Note that Zr-BPT did not adsorb sodium salts of these acids (*e.g.*, sodium benzenesulfonate) (Fig. S10†), clearly indicating that Zr-BPT does not have ion exchange capacity and that these acids are adsorbed as a charge-neutral molecule through some interactions between the molecule and framework (*i.e.*, not through electrostatic interaction between the anion and framework).

**Fig. 5:**
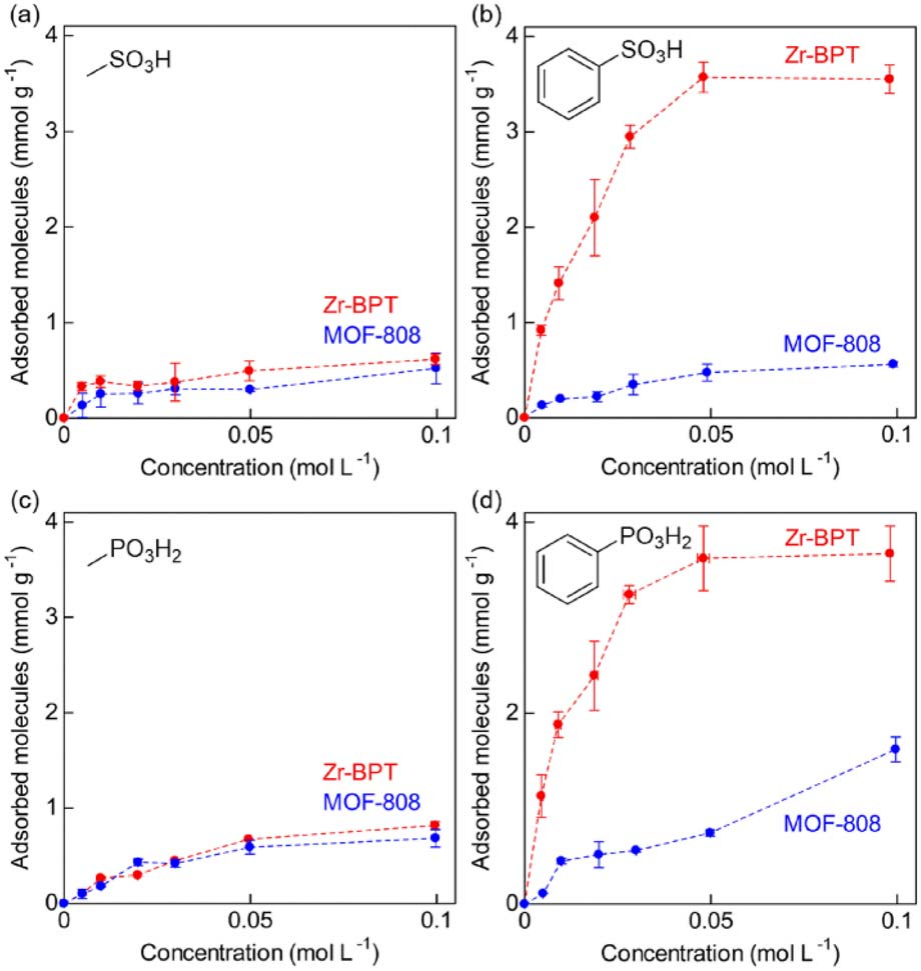
Adsorption isotherms of MOF-808 and Zr-BPT for the strong acid molecules, (a) MS, (b) BS, (c) MP, and (d) PP at 298 K.

In an effort to clarify the selective adsorption property of Zr-BPT for strong acid molecules in more detail, we measured adsorption isotherms for these molecules by measuring the adsorption amount of each guest molecule under various concentrations of aqueous solution at 298 K. For comparison, MOF-808, which is an acid-tolerant Zr-based MOF with large-sized pores (∼18 Å),^30^ was used in the same experiments. The prepared MOF-808 was characterized by XRPD measurements (Fig. S11†). Fig. 5 shows the adsorption isotherms of Zr-BPT and MOF-808 for strong acids, sulfonic acids (benzenesulfonic acid (BS) and methanesulfonic acid (MS)) and phosphonic acids (phenylphosphonic acid (PP) and methylphosphonic acid (MP)). MOF-808 did not exhibit remarkable differences in the adsorption amounts between aryl acids (BS or PP) and alkyl acids (MS or MP); it showed slight adsorption for all of them, regardless of the presence or absence of an aryl group. This indicates that these guest molecules are adsorbed through a weak interaction between the framework and the polar functional group of –SO_3_H or –PO_3_H_2_ in the large pores. By contrast, Zr-BPT showed a significant difference between the adsorption isotherms of aryl acids and alkyl acids. The adsorption amounts for aryl acids (BS and PP) were remarkably higher than those for alkyl acids (MS and MP). This clearly indicated that Zr-BPT exhibits a selective adsorption property for specific guest molecules, even among strong acids. Because the molecular sizes of aryl acids, BS and PP, are apparently larger than those of alkyl acids, MS and MP, this selectivity is not derived from a molecular sieving effect but from a strong affinity for aryl acid molecules. This is the first demonstration of a MOF exhibiting adsorption selectivity among strong acid molecules.

Of note is that there was no apparent change in the XRPD pattern after the acidic guest adsorption (Fig. S12†), indicating that there is no apparent change in the fundamental structure of Zr-BPT during the adsorption process (*i.e.*, Zr-BPT is a second generation compound^31^), which would be due to the rigid Zr-based framework. Regarding the reusability of the MOF, we performed the additional adsorption experiment (2nd use) with the sample that was activated after the adsorption of the aryl acids (1st use). Although we have not yet found the optimal condition for perfect recovery of the initial sample (before 1st use), porous Zr-BPT was recovered (709 m^2^ g^−1^, Fig. S13†) by heating it in DMF at 120 °C for 6 hours. The recovered sample showed apparently lower adsorption amounts of the aryl acids, BS and PP (in the 2nd use), compared with the initial sample (Fig. S14†), suggesting some difference in chemical states. On the other hand, a similar amount of adsorption was observed in the same additional adsorption experiment (3rd use) with the sample that was activated after the 2nd use (under the same conditions (120 °C in DMF for 6 hours)) (Fig. S14†). These results suggest that some irreversible changes occurred during the 1st use. We think that one major reason for this is the formation of defects. As described before, in the Zr-BPT, defects can be formed by exposure to acids due to desorption of the coordinated formate ions. From the ^1^H NMR spectra (Fig. S15†), we found that there is a remarkable decrease of the amount of formate ions (approx. 65% of the formate ions were desorbed) during the 1st use, while almost no change occurs during the 2nd use. The better performance of the initial sample suggests that the included formate ions play an important role in binding the aryl acids. These results clearly indicated that the initial sample is most important and suitable for the aryl acid adsorption, while perfect recovery of the adsorbate might need some chemical treatments.

**Fig. 6:**
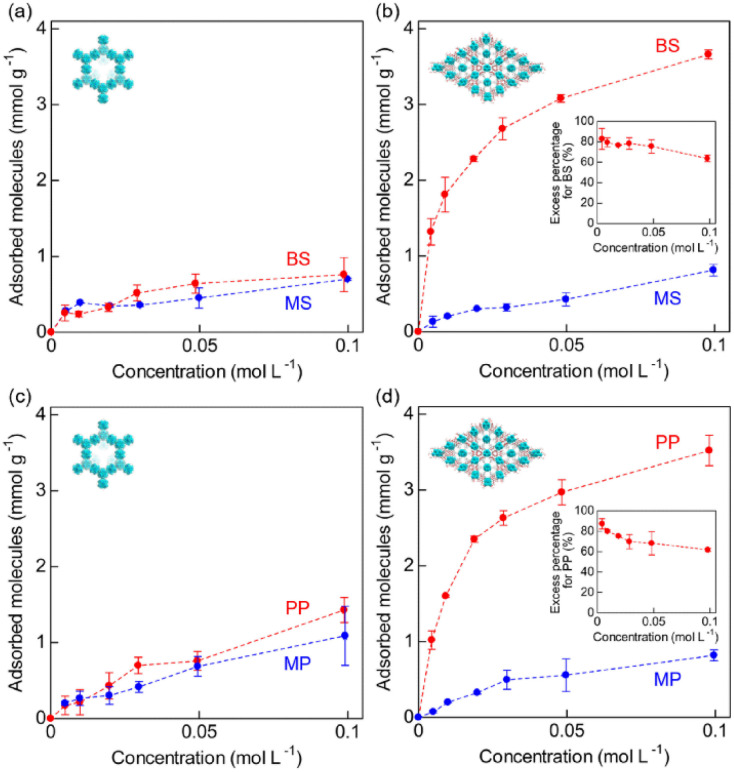
Adsorption amounts of MS and BS by (a) MOF-808 and (b) Zr-BPT from the mixed solution of BS/MS at 298 K. Adsorption amounts of MP and PP by (c) MOF-808 and (d) Zr-BPT from the mixed solution of PP/MP at 298 K. The inset shows the excess percentage for BS or PP in Zr-BPT in the separation experiments.

We also performed separation experiments using mixed solutions of the acids. Fig. 6 shows the results of separation experiments using these mixed solutions: BS/MS or PP/MP. As expected, from the results of adsorption isotherms for each guest, Zr-BPT exhibited remarkably higher adsorption for aryl acids (BS or PP) than for alkyl acids (MS or MP), even in a mixed solution, while MOF-808 showed almost no difference in the adsorption amounts between them. In both cases, for BS and PP, Zr-BPT exhibited high selectivity; if we employed an excess percentage for the aryl acid as an indicator of the selectivity, it was around 60–90% depending on the concentration (Fig. 6), while MOF-808 exhibited almost no excess percentage. This is also the first case of a MOF having an apparent separation ability for strong acids. Considering the difference in structural features between Zr-BPT and MOF-808, the selective adsorption behaviour and separation ability of Zr-BPT is probably due to its small-sized pores that provide an opportunity to bind the guest molecules strongly through multipoint interactions. Unfortunately, we were unable to determine the exact position of the adsorbed guest molecules through Rietveld refinements due to disorder. Nonetheless, we believe that the pore size of Zr-BPT is well matched with specific small molecules, such as these aryl acids, to bind them through additional interactions other than the interaction between the framework and the polar functional group (–SO_3_H or –PO_3_H_2_).

**Fig. 7:**
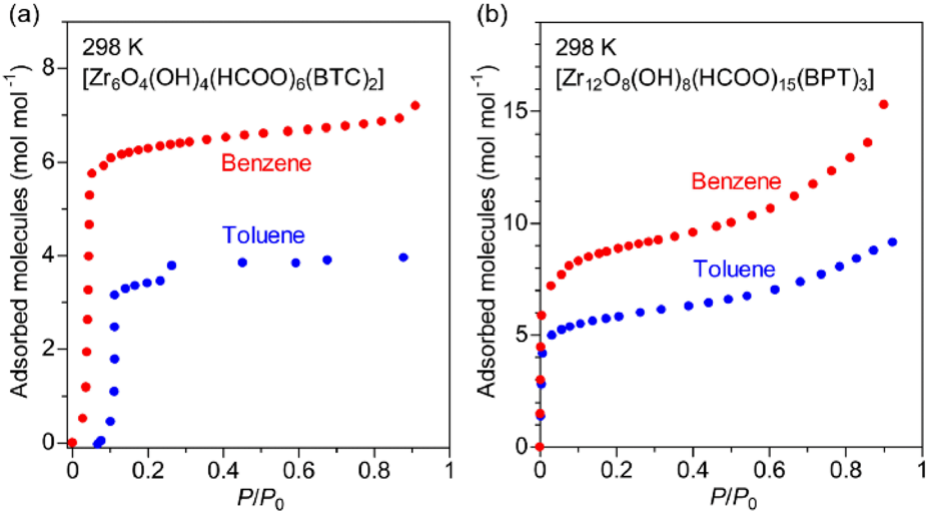
Adsorption isotherms of (a) MOF-808 and (b) Zr-BPT for the vapors of benzene and toluene at 298 K.

To obtain further information about the interaction of Zr-BPT with the aryl acid molecules, we measured adsorption isotherms for the vapors of the guests, benzene and toluene, which have similar molecular structures to BS or PP, except for the absence of the polar functional group of –SO_3_H or –PO_3_H_2_. As shown in Fig. 7, Zr-BPT exhibited a large amount of adsorption for these guests, from a very low-pressure region (<∼0.01 *P*/*P*_0_), indicating a strong binding of these guests. By contrast, MOF-808 adsorbed these guests from a distinctly higher pressure region (<∼0.1 *P*/*P*_0_), indicating a relatively weaker interaction of the framework with them. These results clearly suggest that Zr-BPT has strongly interactive sites for the hydrophobic aryl group in addition to interactive sites for polar functional groups.

We performed additional adsorption experiments using acidic guests with extended alkyl groups. In these experiments, we used carboxylic acids as the guests, due to the unavailability of sulfonic and phosphonic acids with extended alkyl groups. As shown in Fig. S16,†Zr-BPT did not preferably adsorb alkyl acids even in the case with the alkyl-extended guests, while it preferably adsorbed aryl acids as described above (Fig. 4). This clearly suggests that the selective adsorption of aryl acids is not caused by simple hydrophobic interactions but by an interaction specific to the aryl group, for example, π–π interaction or CH–π interaction (otherwise a combination or multiples of them), in addition to the polar interaction between the acidic group and the framework.

To visualize one of the possible adsorption sites in Zr-BPT, we performed Monte Carlo searches using the Adsorption Locator module of Materials Studio.^32,33^ Since it is difficult to determine the position of the defects formed in Zr-BPT during the adsorption experiments, we used non-defective Zr-BPT (*i.e.*, the initial sample) as a structural model for the simulation. As shown in Fig. S17,† we succeeded in obtaining a possible adsorption site in the pore of Zr-BPT, indicating that the void space of Zr-BPT is large enough to adsorb the aryl acid molecule (BS). In the obtained conformation of the adsorbed aryl acid, BS molecule, the aryl group was surrounded by CH groups of the formate ions and BPT ligands, suggesting the opportunity of CH–π interaction between the acid molecule and framework. Although it is difficult to reveal all of the adsorption sites, we think that these CH–π interactions play an important role in the selective adsorption of the aryl acids, which would be related to the excellent adsorption ability of the initial sample (*i.e.*, maximum content of formate ions) for them.

## Conclusions

In conclusion, we succeeded, for the first time, in synthesizing a threefold interpenetrated Zr-based MOF, Zr-BPT. This MOF has the smallest inner micropore (<∼5.6 Å) yet among Zr-based MOFs, with both high acid tolerance and apparent porosity. Employing Zr-BPT, we demonstrated the selective adsorption property of the MOF for specific strong acid molecules and the separation ability for strong acids. Zr-BPT selectively adsorbs aryl acids rather than alkyl acids, probably because of π–π or CH–π interaction with the aryl group in addition to the polar interaction with the acidic group. This could be considered an important example of a new class of separating materials for acid molecules. We also believe that this new MOF has the potential to offer a new platform for separating materials for general (neutral) small guest molecules due to its nature of multipoint contacts with them, although more detailed studies are still underway.

## Data availability

All the data in this study are provided in the main text and ESI.†

## Author contributions

KS and MS designed the project and MS supervised this work. KS carried out the experimental work. MS and KK performed the synchrotron XRPD measurements. KO and KK analyzed the crystal structure. KS and MS carried out computational simulation (Monte Carlo searches). KS and MS prepared the manuscript. All authors contributed to discussions throughout the project and the final editing of the manuscript.

## Conflicts of interest

There are no conflicts to declare.

## Supplementary Material

SC-015-D3SC04171A-s001

SC-015-D3SC04171A-s002
