# Supplementary material for: A novel threefold interpenetrated zirconium metal–organic framework exhibiting separation ability for strong acids
Source: Chem Sci. 2024 Jan 2;15(4):1441–8. doi: 10.1039/d3sc04171a (PMC10806781; doi:10.1039/d3sc04171a)
Supplement: SC-015-D3SC04171A-s001 [file SC-015-D3SC04171A-s001.pdf]

Electronic Supplementary Material (ESI) for Chemical Science.
This journal is © The Royal Society of Chemistry 2024

Supporting Information

A Novel Threefold Interpenetrated Zirconium Metal–Organic Framework Exhibiting Separation Ability for Strong Acids

Kyoko Shiraishi,† Kazuya Otsubo,‡ Kenichi Kato,§ Masaaki Sadakiyo*,†

†*Department of Applied Chemistry, Faculty of Science Division I, Tokyo University of Science, 1-3 Kagurazaka, Shinjuku-ku, Tokyo 162-8601, Japan.*

‡*Department of Chemistry, Faculty of Science Division I, Tokyo University of Science, 1-3 Kagurazaka, Shinjuku-ku, Tokyo 162-8601, Japan.*

§*RIKEN SPring-8 Center, Sayo-gun, Hyogo 679-5148, Japan.*

E-mail: sadakiyo@rs.tus.ac.jp

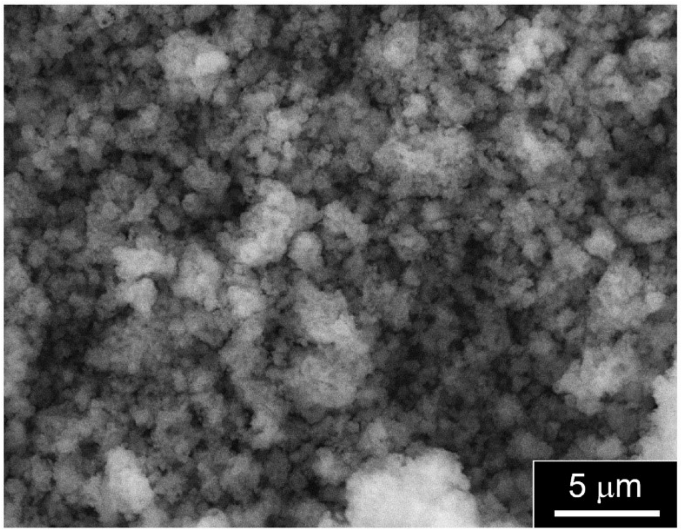

Figure S1. A SEM image of **Zr-BPT**.

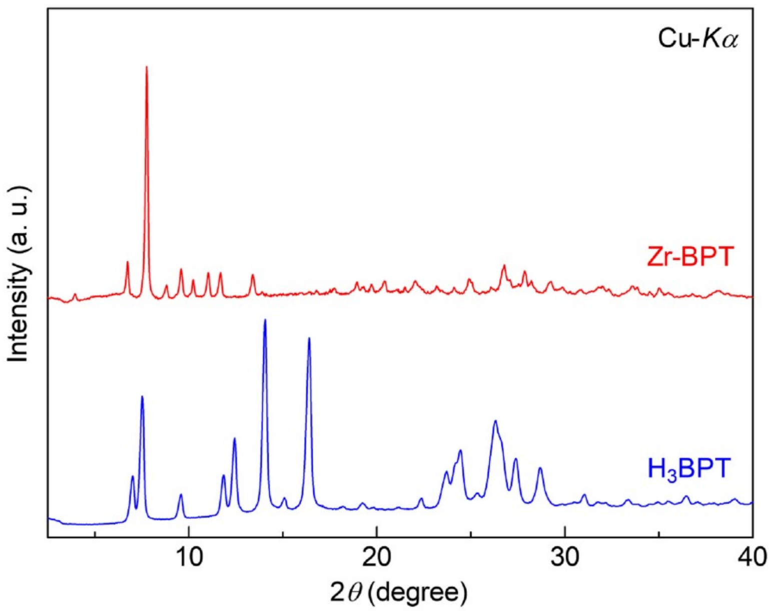

Figure S2. XRPD patterns of **Zr-BPT** and the ligand H_3BPT.

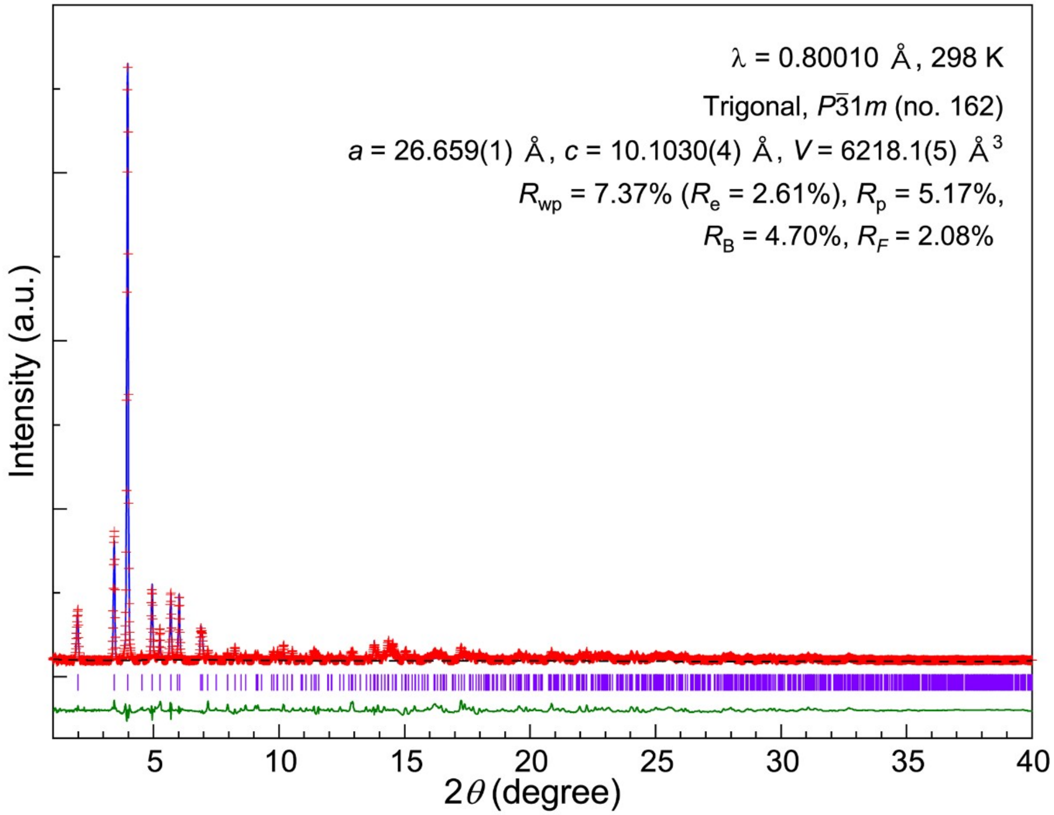

Figure S3. Final Rietveld plots of **Zr-BPT**.

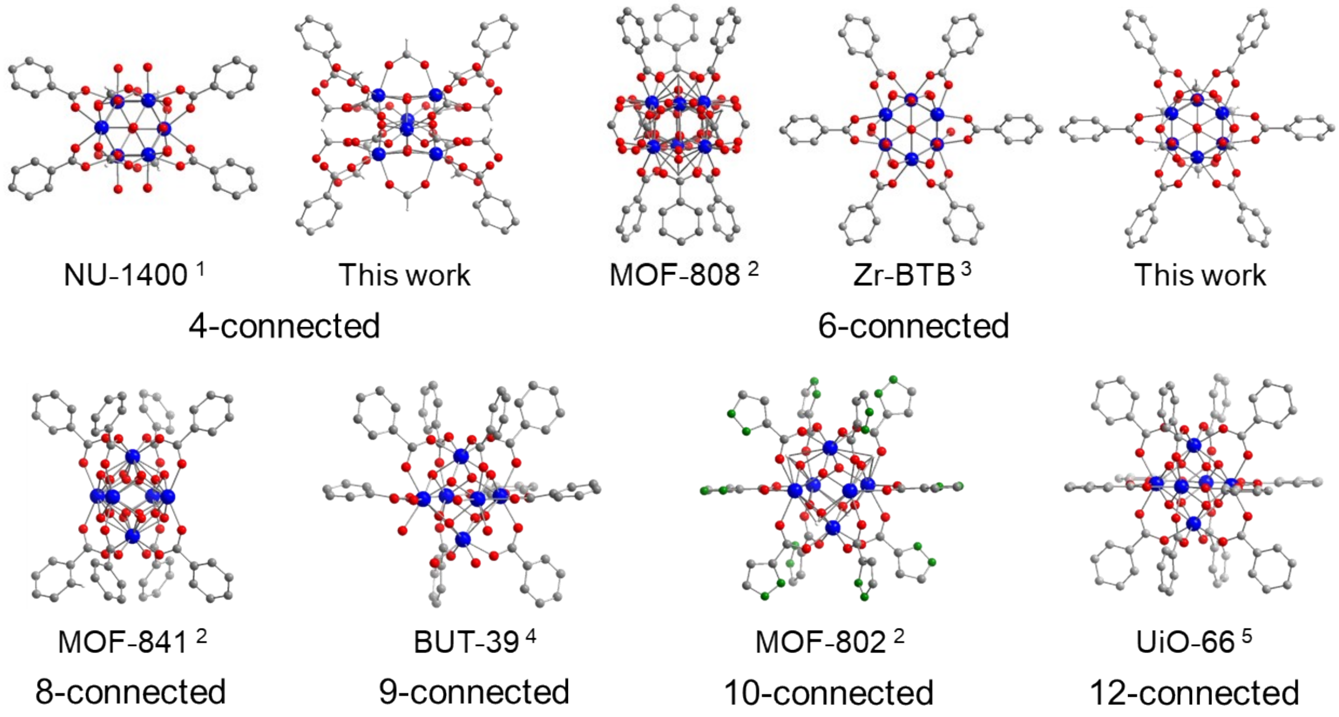

Figure S4. Detailed structures of 4- or 6-connected **Zr_6** clusters in **Zr-BPT** and a structural comparison with various **Zr_6** clusters.

(Reference)

(1) Y. Zhang, X. Zhang, J. Lyu, K. Otake, X. Wang, L. R. Redfern, C. D. Malliakas, Z. Li, T. Islamoglu, B. Wang, O. K. Farha, *J. Am. Chem. Soc.*, 2018, **140**, 11179–11183.

(2) H. Furukawa, F. Gándara, Y.-B. Zhang, J. Jiang, W. L. Queen, M. R. Hudson, O. M. Yaghi, *J. Am. Chem. Soc.*, 2014, **136**, 4369–4381.

(3) R. Wang, Z. Wang, Y. Xu, F. Dai, L. Zhang, D. Sun, *Inorg. Chem.*, 2014, **53**, 7086–7088.

(4) T. He, Y.-Z. Zhang, X.-J. Kong, J. Yu, X.-L. Lv, Y. Wu, Z.-J. Guo, J.-R. Li, *ACS Appl. Mater. Interfaces*, 2018, **10**, 16650–16659.

(5) J. H. Cavka, S. Jakobsen, U. Olsbye, N. Guillou, C. Lamberti, S. Bordiga, K. P. Lillerud, *J. Am. Chem. Soc.*, 2008, **130**, 13850–13851.

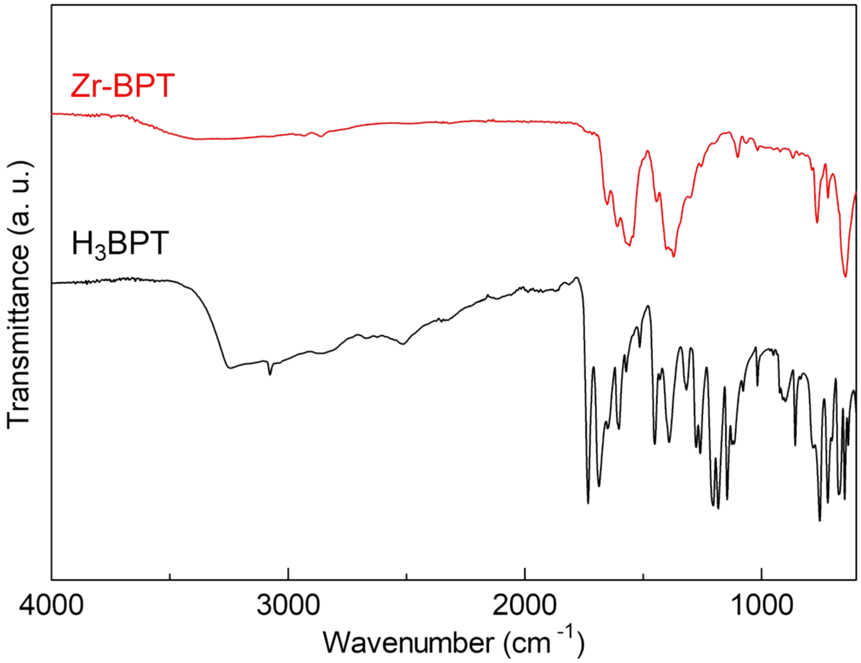

Figure S5. IR spectra of (red) **Zr-BPT** and (black) H_3BPT.

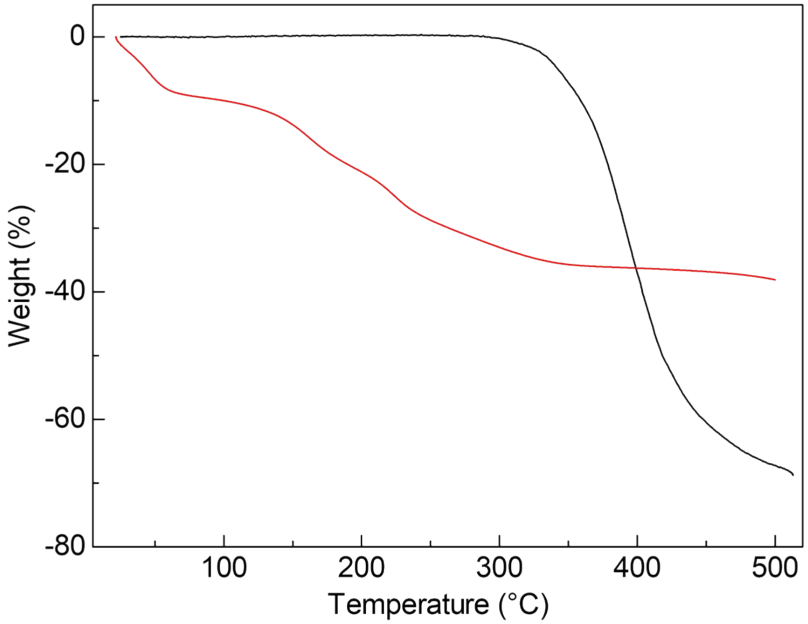

Figure S6. TG curves of (red) **Zr-BPT** and (black) H_3BPT.

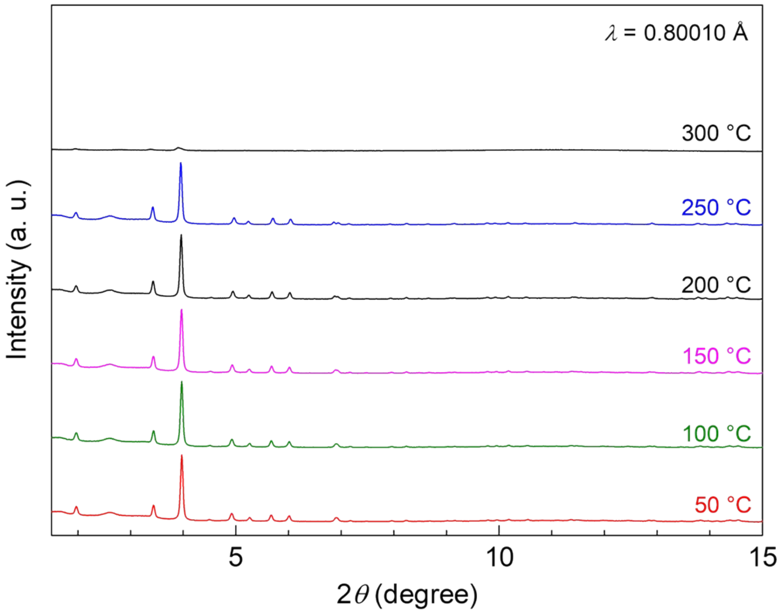

Figure S7. XRPD patterns of **Zr-BPT** (under vacuum after dehydration at 130 °C) at various temperature.

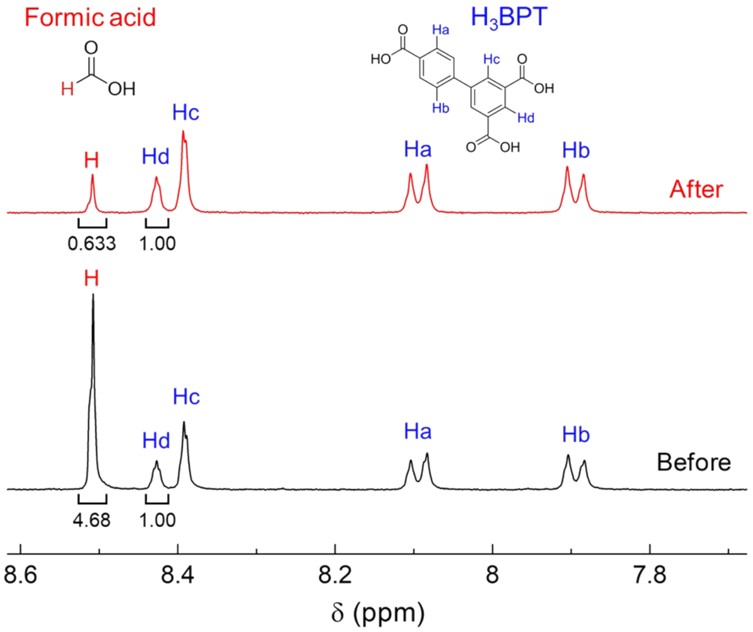

Figure S8. ^{1}H NMR spectra of digested samples of **Zr-BPT** ((black) before and (red) after the exposure to the acid solution (HClaq, pH = 0)). The values of normalized peak area are shown below the spectra.

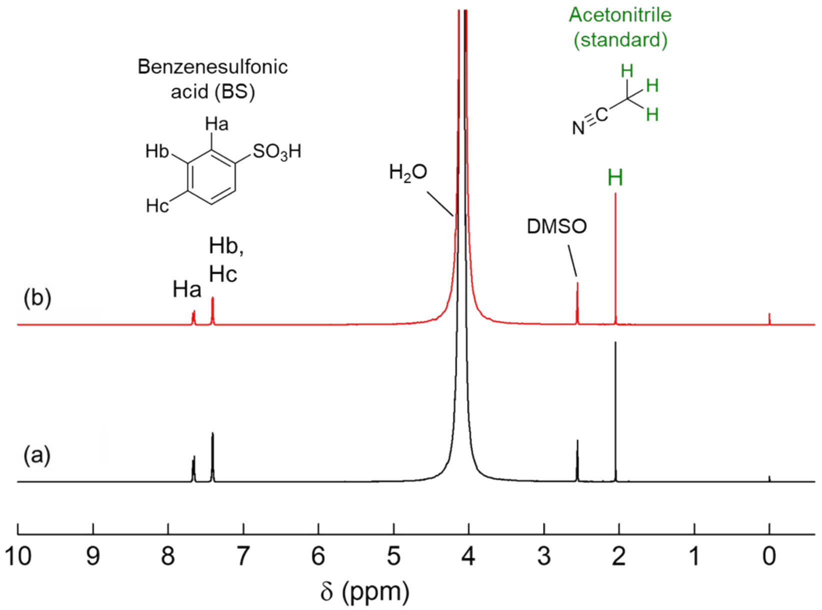

Figure S9. ^{1}H NMR spectra used for acid adsorption experiments, exemplified by BS adsorption ((a) initial solution (before adsorption) and (b) solution after the adsorption).

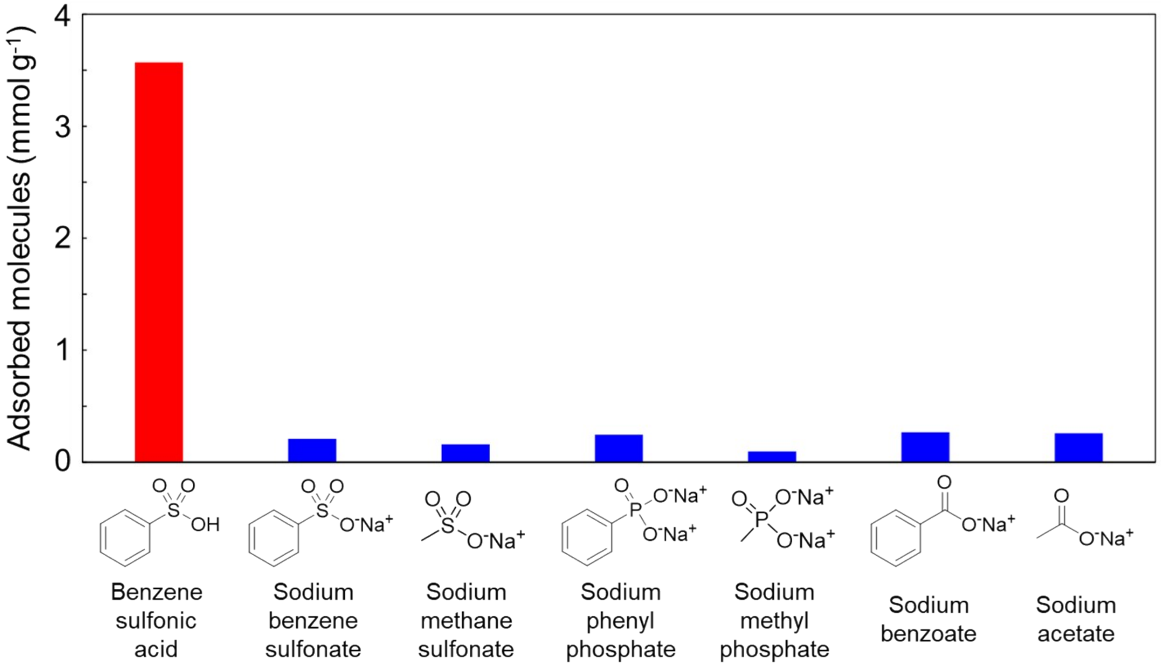

Figure S10. Amounts of adsorbed species in **Zr-BPT** at 298 K (experimental conditions are the same to Figure 4).

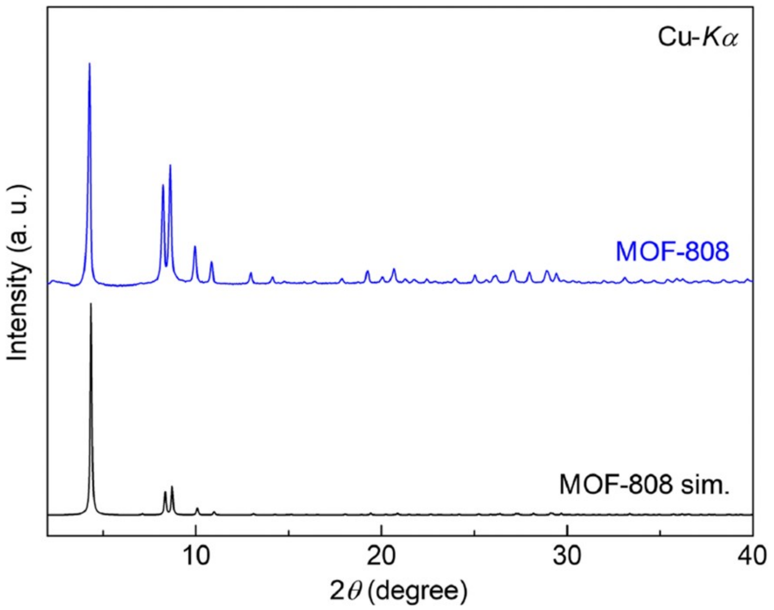

Figure S11. XRPD patterns of the prepared MOF-808.

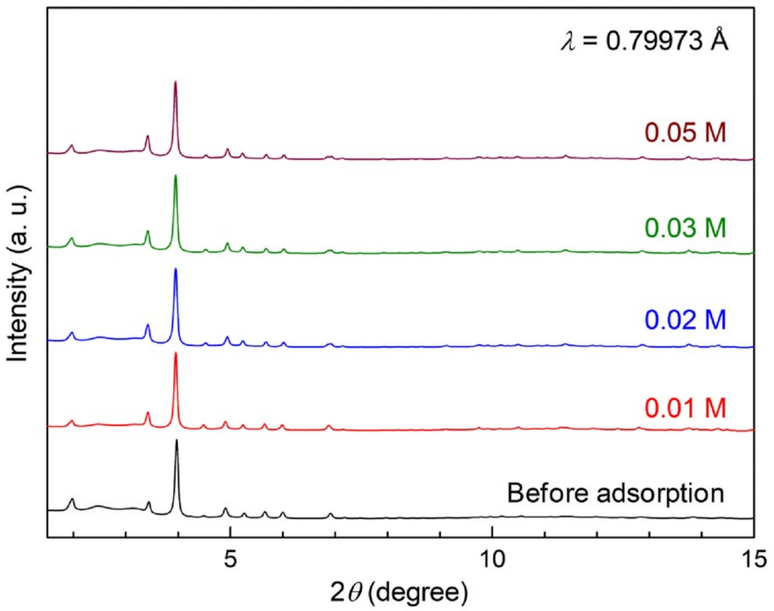

Figure S12. XRPD patterns of **Zr-BPT** before and after the adsorption experiments with various acidic solutions (0.01–0.05 M BS).

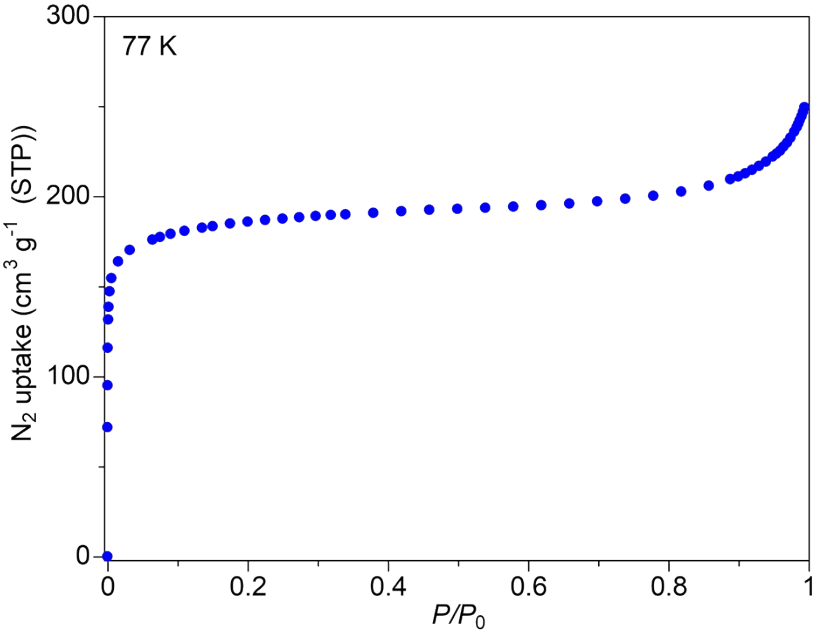

Figure S13. N_2 adsorption isotherms (77 K) of **Zr-BPT** that was recovered (by heating in DMF at 120 °C for 6 hours) after the adsorption of BS.

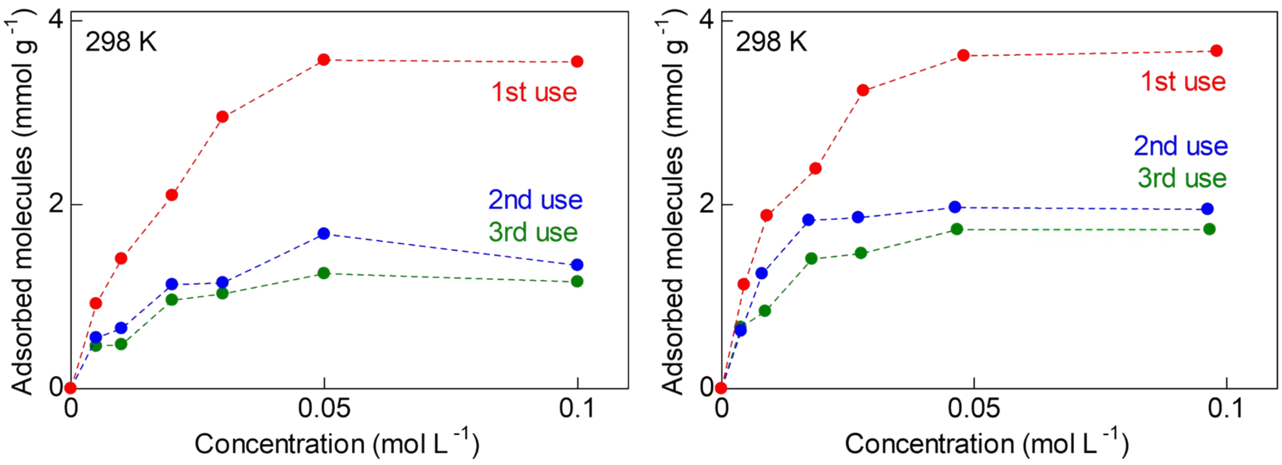

Figure S14. Adsorption isotherms of **Zr-BPT** ((red) 1st use) and recovered **Zr-BPT** ((blue) 2nd and (green) 3rd use.) for (left) BS and (right) PP at 298 K.

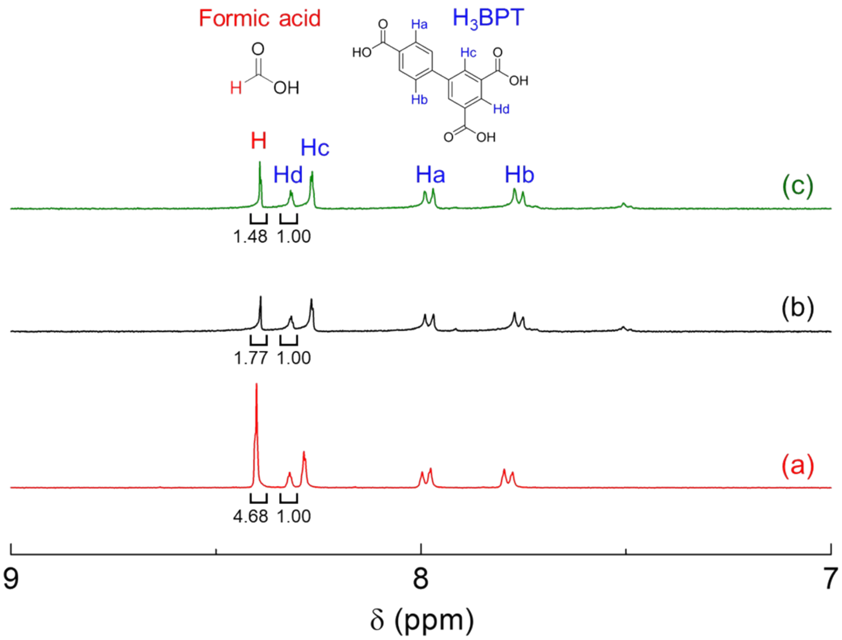

Figure S15. ^{1}H NMR spectra of digested samples of **Zr-BPT** ((a) before the adsorption experiment, (b) the recovered sample after the 1st use for BS, and (c) the re-recovered sample after 2nd use for BS.).

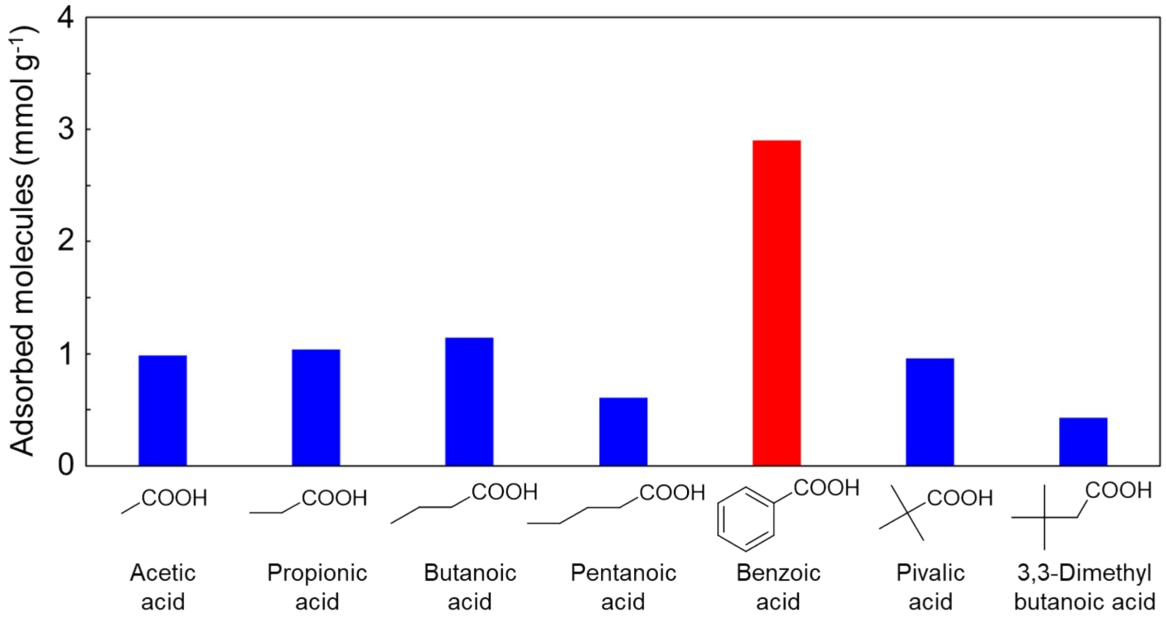

Figure S16. Comparison of adsorption amounts of carboxylic acids (blue: alkyl acid, red: aryl acid) with various functional groups, including different alkyl chains, by **Zr-BPT**. (Experimental conditions are as used for the screening of acid adsorption, Figure 2.)

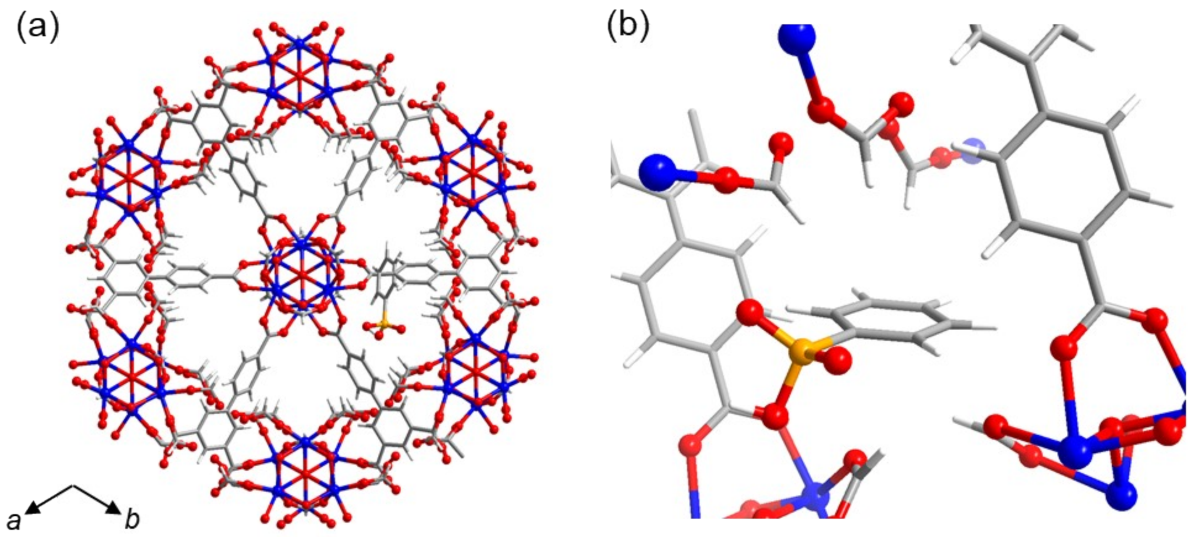

Figure S17. Representation of a possible adsorption site for BS, identified by Adsorption Locator. (a) The view along *c*-axis and (b) magnified view around the adsorbed BS molecule.
